# Comparison of clinical rating scales in genetic frontotemporal dementia within the GENFI cohort

**DOI:** 10.1136/jnnp-2021-326868

**Published:** 2021-08-05

**Authors:** Georgia Peakman, Lucy L Russell, Rhian S Convery, Jennifer M Nicholas, John C Van Swieten, Lize C Jiskoot, Fermin Moreno, Raquel Sanchez-Valle, Robert Laforce, Caroline Graff, Mario Masellis, Maria Carmela Tartaglia, James B Rowe, Barbara Borroni, Elizabeth Finger, Matthis Synofzik, Daniela Galimberti, Rik Vandenberghe, Alexandre de Mendonça, Chris R Butler, Alex Gerhard, Simon Ducharme, Isabelle Le Ber, Fabrizio Tagliavini, Isabel Santana, Florence Pasquier, Johannes Levin, Adrian Danek, Markus Otto, Sandro Sorbi, Jonathan D Rohrer, Genetic FTD Initiative (GENFI), Sónia Afonso

**Affiliations:** 1 Department of Neurodegenerative Disease, University College London Dementia Research Centre, London, UK; 2 Department of Medical Statistics, London School of Hygiene & Tropical Medicine, London, UK; 3 Department of Neurology, Erasmus Medical Center, Rotterdam, The Netherlands; 4 Cognitive Disorders Unit, Department of Neurology, Hospital Universitario de Donostia, San Sebastian, Spain; 5 Neuroscience Area, Biodonostia Health Research Institute, Donostia-san Sebastian, Spain; 6 Alzheimer’s Disease and Other Cognitive Disorders Unit, Neurology Service, Hospital Clínic, Institut d’Investigacións Biomèdiques August Pi I Sunyer, University of Barcelona, Barcelona, Spain; 7 Clinique Interdisciplinaire de Mémoire, Département des Sciences Neurologiques, CHU de Québec, and Faculté de Médecine, Laval University, Quebec, Quebec, Canada; 8 Center for Alzheimer Research, Division of Neurogeriatrics, Department of Neurobiology, Care Sciences and Society, Bioclinicum, Karolinska Institutet, Stockholm, Sweden; 9 Unit for Hereditary Dementias, Theme Aging, Karolinska University Hospital, Stockholm, Sweden; 10 Sunnybrook Health Sciences Centre, Sunnybrook Research Institute, University of Toronto, Toronto, Ontario, Canada; 11 Tanz Centre for Research in Neurodegenerative Diseases, University of Toronto, Toronto, Ontario, Canada; 12 Department of Clinical Neurosciences, University of Cambridge, Cambridge, UK; 13 Neurology Unit, Department of Clinical and Experimental Sciences, University of Brescia, Brescia, Italy; 14 Clinical Neurological Sciences, University of Western Ontario, London, Ontario, Canada; 15 Dept. of Neurodegenerative Diseases, Eberhard Karls University Tubingen Hertie Institute for Clinical Brain Research, Tubingen, Germany; 16 Center for Neurodegenerative Diseases, DZNE, Tübingen, Germany; 17 Fondazione IRCCS Ca' Granda Ospedale Maggiore Policlinico, Milan, Italy; 18 Centro Dino Ferrari, University of Milan, Milan, Italy; 19 Laboratory for Cognitive Neurology, Department of Neurosciences, KU Leuven, Leuven, Belgium; 20 Neurology Service, KU Leuven University Hospitals Leuven, Leuven, Belgium; 21 Leuven Brain Institute, KU Leuven, Leuven, Belgium; 22 Faculty of Medicine, University of Lisbon, Lisbon, Portugal; 23 Nuffield Department of Clinical Neurosciences, University of Oxford Medical Sciences Division, Oxford, UK; 24 Department of Brain Sciences, Imperial College London, London, UK; 25 Division of Neuroscience and Experimental Psychology, Wolfson Molecular Imaging Centre, The University of Manchester, Manchester, UK; 26 Departments of Geriatric Medicine and Nuclear Medicine, University of Duisburg-Essen, Duisburg, Germany; 27 Department of Psychiatry, McGill University Health Centre, Montreal, Québec, Canada; 28 McConnell Brain Imaging Centre, Montreal Neurological Institute and Hospital, Montreal, Québec, Canada; 29 Sorbonne Université, Paris Brain Institute – Institut du Cerveau – ICM, Inserm U1127, CNRS UMR 7225, Hôpital Universitaire Pitié Salpêtrière, Paris, France; 30 Centre de référence des démences rares ou précoces, IM2A, Département de Neurologie, Hôpital Universitaire Pitié Salpêtrière, Paris, France; 31 Départment de Neurologie, AP-HP - Hôpital Pitié-Salpêtrière, Paris, France; 32 Fondazione IRCCS Istituto Neurologico Carlo Besta, Milano, Italy; 33 University Hospital of Coimbra (HUC), Neurology Service, University of Coimbra Faculty of Medicine, Coimbra, Portugal; 34 Center for Neuroscience and Cell Biology, University of Coimbra Faculty of Medicine, Coimbra, Portugal; 35 University of Lille, Lille, France; 36 CNR-MAJ, Labex Distalz, LiCEND Lille, CHU Lille, Lille, France; 37 Inserm 1172, Lille, France; 38 Department of Neurology, Ludwig-Maximilians-Universität München, Munchen, Germany; 39 German Center for Neurodegenerative Diseases, DZNE, Munich, Germany; 40 Munich Cluster of Systems Neurology (SyNergy), Munich, Germany; 41 Department of Neurology, University of Ulm, Ulm, Germany; 42 Department of Neurofarba, University of Florence, Firenze, Italy; 43 IRCCS Fondazione Don Carlo Gnocchi, Firenze, Italy

**Keywords:** frontotemporal dementia

## Abstract

**Background:**

Therapeutic trials are now underway in genetic forms of frontotemporal dementia (FTD) but clinical outcome measures are limited. The two most commonly used measures, the Clinical Dementia Rating (CDR)+National Alzheimer’s Disease Coordinating Center (NACC) Frontotemporal Lobar Degeneration (FTLD) and the FTD Rating Scale (FRS), have yet to be compared in detail in the genetic forms of FTD.

**Methods:**

The CDR+NACC FTLD and FRS were assessed cross-sectionally in 725 consecutively recruited participants from the Genetic FTD Initiative: 457 mutation carriers (77 microtubule-associated protein tau (*MAPT)*, 187 *GRN*, 193 *C9orf72*) and 268 family members without mutations (non-carrier control group). 231 mutation carriers (51 *MAPT,* 92 *GRN,* 88 *C9orf72*) and 145 non-carriers had available longitudinal data at a follow-up time point.

**Results:**

Cross-sectionally, the mean FRS score was lower in all genetic groups compared with controls: *GRN* mutation carriers mean 83.4 (SD 27.0), *MAPT* mutation carriers 78.2 (28.8), *C9orf72* mutation carriers 71.0 (34.0), controls 96.2 (7.7), p<0.001 for all comparisons, while the mean CDR+NACC FTLD Sum of Boxes was significantly higher in all genetic groups: *GRN* mutation carriers mean 2.6 (5.2), *MAPT* mutation carriers 3.2 (5.6), *C9orf72* mutation carriers 4.2 (6.2), controls 0.2 (0.6), p<0.001 for all comparisons. Mean FRS score decreased and CDR+NACC FTLD Sum of Boxes increased with increasing disease severity within each individual genetic group. FRS and CDR+NACC FTLD Sum of Boxes scores were strongly negatively correlated across all mutation carriers (r_s_=−0.77, p<0.001) and within each genetic group (r_s_=−0.67 to −0.81, p<0.001 in each group). Nonetheless, discrepancies in disease staging were seen between the scales, and with each scale and clinician-judged symptomatic status. Longitudinally, annualised change in both FRS and CDR+NACC FTLD Sum of Boxes scores initially increased with disease severity level before decreasing in those with the most severe disease: controls −0.1 (6.0) for FRS, −0.1 (0.4) for CDR+NACC FTLD Sum of Boxes, asymptomatic mutation carriers −0.5 (8.2), 0.2 (0.9), prodromal disease −2.3 (9.9), 0.6 (2.7), mild disease −10.2 (18.6), 3.0 (4.1), moderate disease −9.6 (16.6), 4.4 (4.0), severe disease −2.7 (8.3), 1.7 (3.3). Sample sizes were calculated for a trial of prodromal mutation carriers: over 180 participants per arm would be needed to detect a moderate sized effect (30%) for both outcome measures, with sample sizes lower for the FRS.

**Conclusions:**

Both the FRS and CDR+NACC FTLD measure disease severity in genetic FTD mutation carriers throughout the timeline of their disease, although the FRS may be preferable as an outcome measure. However, neither address a number of key symptoms in the FTD spectrum, for example, motor and neuropsychiatric deficits, which future scales will need to incorporate.

## Introduction

Frontotemporal dementia (FTD) is a spectrum of heterogenous disorders characterised by neurodegeneration of the frontal and temporal lobes. A total of 20%–30% of FTD cases are genetic,^1 2^ with the majority caused by autosomal dominant mutations in three genes^3^: chromosome 9 open reading frame 72 (*C9orf72*),^4^ progranulin (*GRN*)^5^ and microtubule-associated protein tau (*MAPT*).^6^ Clinical syndromes span changes in behaviour (behavioural variant FTD, bvFTD),^7^ language (primary progressive aphasia, PPA)^8^ and motor function (progressive supranuclear palsy, PSP, corticobasal syndrome, CBS and FTD with amyotrophic lateral sclerosis, FTD-ALS).^9–11^ Age of symptom onset, and disease progression and duration vary between and within genetic groups.^12^

The ability to accurately evaluate disease stage and track clinical change in FTD across the spectrum of phenotypes is critical for the design of future trials of disease-modifying therapies. Two candidate global severity measures specific to FTD are the Clinical Dementia Rating (CDR) Dementia Staging Instrument and the FTD Rating Scale (FRS). The CDR is a widely used scale that was developed to stage the severity of dementia in the Alzheimer’s Disease spectrum.^13 14^ Impairment in six cognitive and functional domains are assessed by a neurologist through semistructured interview with both the patient and caregiver. The CDR was extended for FTD by introducing a behaviour and a language domain, taken from the National Alzheimer’s Disease Coordinating Centre (NACC) Frontotemporal Lobar Degeneration (FTLD) module (CDR+NACC FTLD).^15 16^ A version of the global CDR scoring system^17^ (without the emphasis on the memory domain) has been developed to apply to the CDR+NACC FTLD, which classifies cases into five severity levels based on the number and severity of the ratings given for the eight domains.^18^ The CDR+NACC FTLD has shown ability to detect mild to severe symptoms in sporadic and genetic FTD cohorts^15 16 18 19^ and capture disease progression over 1–2 years.^15 20^ The FRS is a 30-item caregiver questionnaire developed with the aim of staging FTD severity based on behavioural changes and functional decline.^21^ The scale captures six levels of impairment from very mild to profound. Disease severity according to the FRS has been found to correlate with the CDR^21 22^ and CDR+NACC-FTLD,^23^ but a detailed evaluation of the measure across the range of presymptomatic and symptomatic FTD has not been reported.

Few studies have directly compared the FRS and CDR+NACC FTLD staging tools, particularly in relation to the increasingly used CDR+NACC FTLD global scoring system. The objectives of this study were to: (1) evaluate and compare how the FRS and CDR+NACC FTLD scales characterise disease stage and severity in the spectrum of presymptomatic and symptomatic genetic FTD, using cross-sectional data from the Genetic FTD Initiative (GENFI) cohort; (2) examine and compare longitudinal change in the scales using data at 1-year follow-up and (3) estimate the sample sizes required to detect a small or moderate size effect on disease progression based on the two candidate outcome measures.

## Methods

### Cohort

From the fifth data freeze of the GENFI study, 725 participants with both FRS and CDR+NACC FTLD data available for at least one time point were included in the study: 457 mutation carriers (77 *MAPT*, 187 *GRN*, 193 *C9orf72*) and 268 family members without mutations (non-carrier control group).

### Measures

All participants underwent a standardised history and examination including the Mini-Mental State Examination (MMSE), with symptomatic status judged by the assessing clinician according to consensus diagnostic criteria.

#### Frontotemporal dementia Rating Scale (FRS)

The FRS is a 30-item questionnaire covering seven areas: behaviour, outing and shopping, household chores and telephone, finances, medications, meal preparation and eating, and self care and mobility. The FRS was completed by an informant (family member or caregiver) by rating the frequency of difficulties in these areas (‘all the time’, ‘sometimes’, ‘never’). Raw scores are converted to a percentage (total number of ‘never’ responses/total number of applicable questions) to exclude any items that were not applicable to the patient. Lower percentage scores therefore denote greater impairment of everyday abilities and behavioural change. In the original development of the scale, six severity stages were identified and operationalised in 75 patients with FTD (very mild, 100%–97%; mild, 96%–80%; moderate, 79%–41%; severe, 40%–13%; very severe, 12%–3%; profound, 2%–0%).^21^ One modification was made to these classifications for use in the GENFI cohort because the FRS is also collected on non-carrier family members: a score of 100% was considered ‘asymptomatic’ rather than ‘very mild’. The ‘very mild’ category in this study therefore encompasses scores of 97%–99% instead of 97%–100%.

#### CDR+NACC-FTLD

The eight domains of the CDR+NACC FTLD assess memory, orientation, judgement and problem solving, community affairs, home and hobbies, personal care, overall behaviour and overall language. Based on a semistructured interview with the patient and an informant, the presence of impairment in each of these domains is rated by a clinician using scores of 0 (absent), 0.5 (questionable/very mild), 1 (mild), 2 (moderate) and 3 (severe).^15–17^ The sum of boxes score (CDR+NACC-FTLD-SB) is calculated by summing the ratings given for the eight domains. Thus, a higher sum of boxes value denotes greater symptomatology. The CDR+NACC FTLD global rating was determined using the published scoring rules,^18^ whereby a rating on a five-point scale is given (0, 0.5, 1, 2, 3) based on the severity of the ratings given for the eight domains. All eight domains are given equal weighting when calculating the global score, so it does not relate to a specific FTD variant, and if any domain has a rating above 0 then the global score is at least 0.5. Therefore, cases with no impairment in any domain are given a global rating of 0, those with mild cognitive, behavioural or language impairment are rated 0.5, those with mild but definite symptomology are intended to receive a rating of 1, those with moderate dementia 2 and severe dementia 3. Global ratings can be reduced into three broader disease severity levels: normal or asymptomatic (0), very mild or prodromal cognitive, behavioural or language impairment (0.5) and fully symptomatic (≥1).^18^

### Statistical analysis

#### Descriptive statistics and group comparisons

Data were analysed using SPSS V.26 or STATA V.16. Demographic variables were compared between groups using independent sample t-tests or Mann-Whitney U tests when n<30. Sex was compared between groups using χ^2^ tests. A linear regression model was used to compare both FRS and CDR+NACC-FTLD-SB scores between groups; bootstrapping with 1000 repetitions was used for data that were not normally distributed. Correlations between FRS percentage score and CDR+NACC FTLD Sum of Boxes scores were generated using Spearman rank correlation coefficients (two-tailed), as were correlations of both scales with disease duration (years since clinician-judged symptom onset – analysis restricted to symptomatic participants) and MMSE score.

#### Longitudinal analyses

Of the baseline sample, 231 mutation carriers (51 *MAPT,* 92 *GRN,* 88 *C9orf72*) and 145 non-carriers had FRS and CDR+NACC FTLD data available at a follow-up time point. Mean time between baseline and follow-up was 1.3 years (SD=0.5). For both scales, annualised change was calculated as: [follow-up score] - [baseline score]/time between baseline and follow-up. Annualised change was compared between the mutation carrier group and controls using a linear regression model; bootstrapping with 1000 repetitions was used for data that were not normally distributed.

#### Sample size calculation

To explore the use of the FRS and CDR+NACC-FTLD-SB scores as potential outcome measures in treatment trials, sample sizes per arm of a two-arm trial of a disease modifying therapy (with 1:1 randomisation to placebo vs active treatment) were calculated using an analysis of covariance method. The analysis focused on mutation carriers with a baseline CDR+NACC FTLD global rating of 0.5 (ie, a prodromal trial), with the desired treatment effect hypothesised as a reduction in progression from the mean score of the outcome measure in the global 0.5 CDR+NACC FTLD group to the mean score of the outcome measure in the global 1 CDR+NACC FTLD group, that is, slowing of progression from prodromal to fully symptomatic. The following formula was used:

*ρ* is the correlation between baseline and follow-up scores of the outcome measure in mutation carriers, *σ* is the SD of scores at follow-up, *δ* is the treatment effect (difference in mean score between the prodromal (0.5) group and mild symptomatic (1) group), α is the significance level, set at 0.05. and 1- β is the power to detect a treatment effect, set at β=0.2 ie,that is, power 80%.

## Results

### Demographics

The demographic and clinical characteristics of the participants in each genetic group at their baseline time point are summarised in table 1. The groups shared similar demographic profiles, except that the *MAPT* mutation carriers and the controls were younger than the *C9orf72* mutation carriers (*MAPT* t=−3.207, p=0.002; controls t=−4.030, p<0.001) and *GRN* mutation carriers (*MAPT* t=−2.875, p=0.004; controls t=−3.501, p=0.001).

**Table 1:** Baseline demographic and clinical characteristics of study cohort by genetic group

	GRN mutation carriers	MAPT mutation carriers	C9orf72 mutation carriers	Controls
N	187	77	193	268
Age (years)	50.8 (13.6)	45.5 (13.8)	51.5 (13.8)	46.4 (12.9)
Sex (% female)	60.4	54.5	50.8	58.6
Education (years)	14.0 (3.7)	14.1 (3.4)	13.9 (3.3)	14.5 (3.3)
CDR				
CDR Sum of Boxes	1.9 (4.1)	2.4 (4.4)	3.1 (4.9)	0.1 (0.4)
CDR+NACC FTLD Sum of Boxes	2.6 (5.2)	3.2 (5.6)	4.2 (6.2)	0.2 (0.6)
CDR+NACC-FTLD-Global (% of participants)				
0	61.0	54.5	47.7	80.2
0.5	13.4	16.9	16.6	17.5
1	10.2	9.1	9.3	2.2
2	8.0	10.4	13.5	0.0
3	7.5	9.1	13.0	0.0
FRS				
Percentage score (0–100)	83.4 (27.0)	78.2 (28.8)	71.0 (34.0)	96.2 (7.7)
Severity category (% of participants)				
Asymptomatic	48.1	37.7	29.0	61.6
Very mild	7.5	7.8	6.7	10.8
Mild	19.3	22.1	24.9	23.5
Moderate	13.9	13.0	12.4	4.1
Severe	8.6	15.6	18.1	0.0
Very severe	2.7	3.9	7.8	0.0
Profound	0.0	0.0	1.0	0.0

Values are mean (SD) unless stated.

CDR+NACC FTLD= CDR Dementia Staging Instrument plus Behaviour and Language domains from the NACC FTLD module.

CDR, Clinical Dementia Rating; FRS, Frontotemporal dementia Rating Scale; FTLD, Frontotemporal Lobar Degeneration; MAPT, microtubule-associated protein tau; NACC, National Alzheimer’s Disease Coordinating Center.

Defining disease severity in the mutation carriers by CDR+NACC FTLD global rating, 54.3% were asymptomatic (CDR+NACC FTLD global=0), 15.3% had a prodromal phenotype (0.5) and 30.4% had a symptomatic phenotype (≥1).

### Cross-sectional change in the FRS and CDR+NACC-FTLD

#### Comparison of both FRS and CDR+NACC-FTLD-SB between groups

The mean FRS% score in all genetic groups was lower than controls (p<0.001 for all comparisons): *GRN* mutation carriers mean 83.4 (SD 27.0), *MAPT* mutation carriers 78.2 (28.8), *C9orf72* mutation carriers 71.0 (34.0), controls 96.2 (7.7) (table 1 and online supplemental table 1). There was also a significant difference between the *C9orf72* group and both the *GRN* group (p<0.001) and the *MAPT* group (p=0.032).

10.1136/jnnp-2021-326868.supp1 Supplementary data

The mean CDR+NACC-FTLD-SB score was higher in all genetic groups compared with controls (all p<0.001): *GRN* mutation carriers mean 2.6 (5.2), *MAPT* mutation carriers 3.2 (5.6), *C9orf72* mutation carriers 4.2 (6.2), controls 0.2 (0.6) (table 1, online supplemental table 2). A significant difference was seen between the *C9orf72* and *GRN* groups (p=0.001).

#### Comparison of both FRS and CDR+NACC-FTLD-SB within genetic groups by disease severity

**Figure 1:**
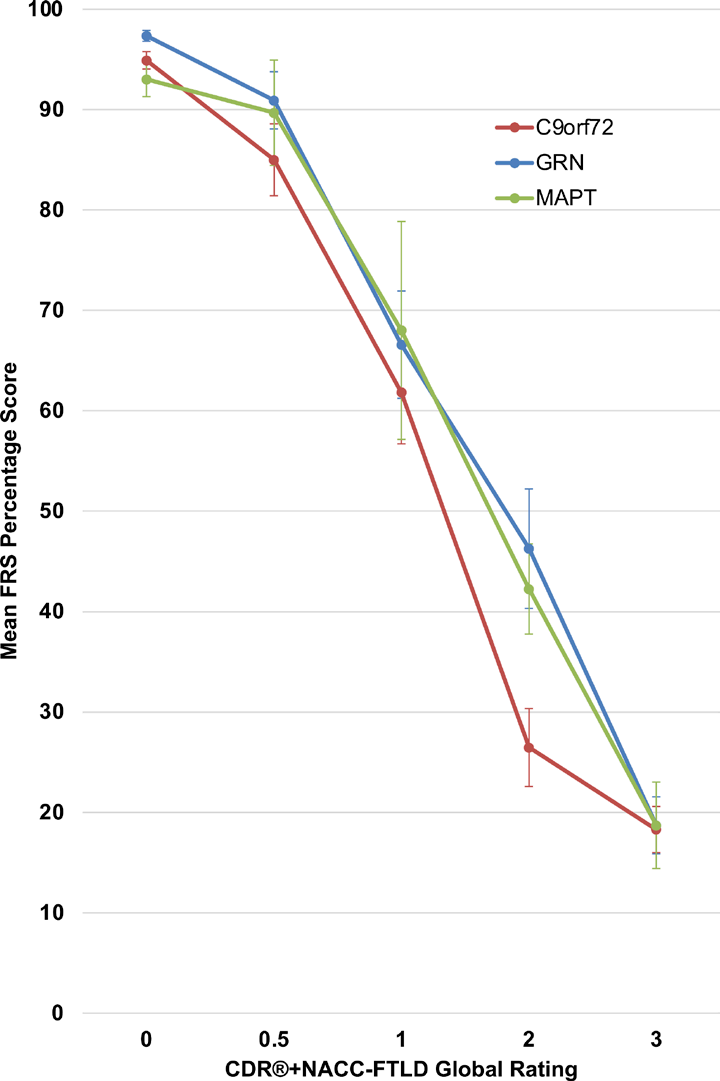
Mean FRS percentage score according to CDR+NACC FTLD global rating in mutation carriers at baseline. Error bars represent SE of the mean. CDR+NACC FTLD global ratings: GRN 0, N=114; 0.5, N=25; 1, N=19; 2, N=15; 3, N=14; MAPT 0, N=42; 0.5, N=13; 1, N=7; 2, N=8; 3, N=7; C9orf72 0, N=92; 0.5, N=32; 1, N=18; 2, N=26; 3, N=25. CDR, Clinical Dementia Rating; FRS, Frontotemporal dementia Rating Scale; NACC, National Alzheimer’s Disease Coordinating Center.

Mean scores on the FRS according to CDR+NACC FTLD severity level (0, 0.5, ≥1) for each genetic group are reported in table 2, and according to individual CDR+NACC FTLD global rating (0–3) are presented in figure 1. *GRN, MAPT* and *C9orf72* mutation carriers with a global rating of 0 had comparable FRS scores to controls (online supplemental table 3). Within both the *GRN* and *C9orf72* mutation carriers, the mean FRS score was significantly lower in cases with a global rating of 0.5 compared with those with 0. Within every genetic group, the cases with a global rating of ≥1 had significantly lower FRS scores than those with 0 or 0.5.

**Table 2:** Baseline FRS scores according to CDR+NACC FTLD severity level, by genetic group

CDR+NACC FTLD Global Group	*GRN* mutation carriers			*MAPT* mutation carriers			*C9orf72* mutation carriers			Controls
	0	0.5	≥1	0	0.5	≥1	0	0.5	≥1	
N	114	25	48	42	13	22	92	32	69	268
FRS % Score	97.4 (5.7)	90.9† (14.2)	46.3*, †, ‡ (28.1)	93.0 (11.2)	89.7 (18.9)	43.0*, †, ‡ (27.1)	94.9 (8.2)	85.0*, † (20.2)	32.7*, †, ‡ (25.0)	96.2 (7.7)
CDR+NACC FTLD Sum of Boxes	0.0 (0.0)	1.0*, † (0.8)	9.7*, †, ‡ (6.2)	0.0 (0.0)	1.1 (0.8)*, †	10.5*, †, ‡ (5.9)	0.0 (0.0)	1.1*, † (0.7)	11.1*, †, ‡ (5.5)	0.2 (0.6)

Values are mean (SD) unless stated.

CDR+NACC FTLD=CDR Dementia Staging Instrument plus Behaviour and Language domains from the NACC FTLD module.

CDR+NACC FTLD sum of boxes scores are also shown in the same groups for comparison.

*P<0.05 versus controls.

†P<0.05 versus CDR+NACC-FTLD Global score=0 group (within the same genetic group).

‡P<0.05 versus CDR+NACC-FTLD Global score=0.5 group (within the same genetic group).

CDR, Clinical Dementia Rating; FRS, Frontotemporal dementia Rating Scale; FTLD, Frontotemporal Lobar Degeneration; NACC, National Alzheimer’s Disease Coordinating Center.

For comparison, mean CDR+NACC-FTLD-SB scores according to severity level for each genetic group are also reported in table 2. The mean CDR+NACC-FTLD-SB scores were higher in those with a global rating of 0.5 and ≥1 than either controls or those with a global rating of 0 in all three genetic groups (online supplemental table 4).

#### Correlation of FRS and CDR+NACC-FTLD-SB

**Figure 2:**
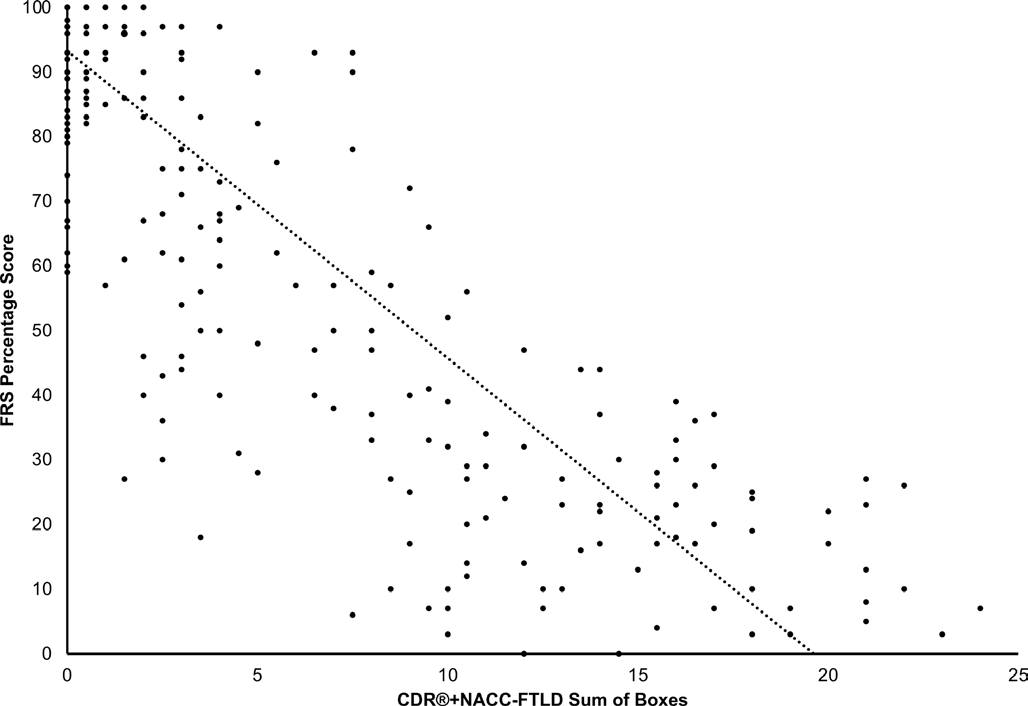
Scatter plot of FRS percentage scores and CDR+NACC FTLD sum of boxes scores in all mutation carriers at baseline. CDR, Clinical Dementia Rating; FRS, Frontotemporal dementia Rating Scale; NACC, National Alzheimer’s Disease Coordinating Center.

In the mutation carriers as a whole, FRS and CDR+NACC-FTLD-SB scores were strongly negatively correlated (r_s_=−0.77, p<0.001) (figure 2). Similar associations were found in the individual genetic groups: *GRN* mutation carriers (r_s_=−0.75, p<0.001); *MAPT* mutation carriers (r_s_=−0.67, p<0.001); *C9orf72* mutation carriers (r_s_=−0.81, p<0.001) (online supplemental figure 1).

#### Comparison of FRS and CDR+NACC-FTLD-SB by severity categories

**Figure 3:**
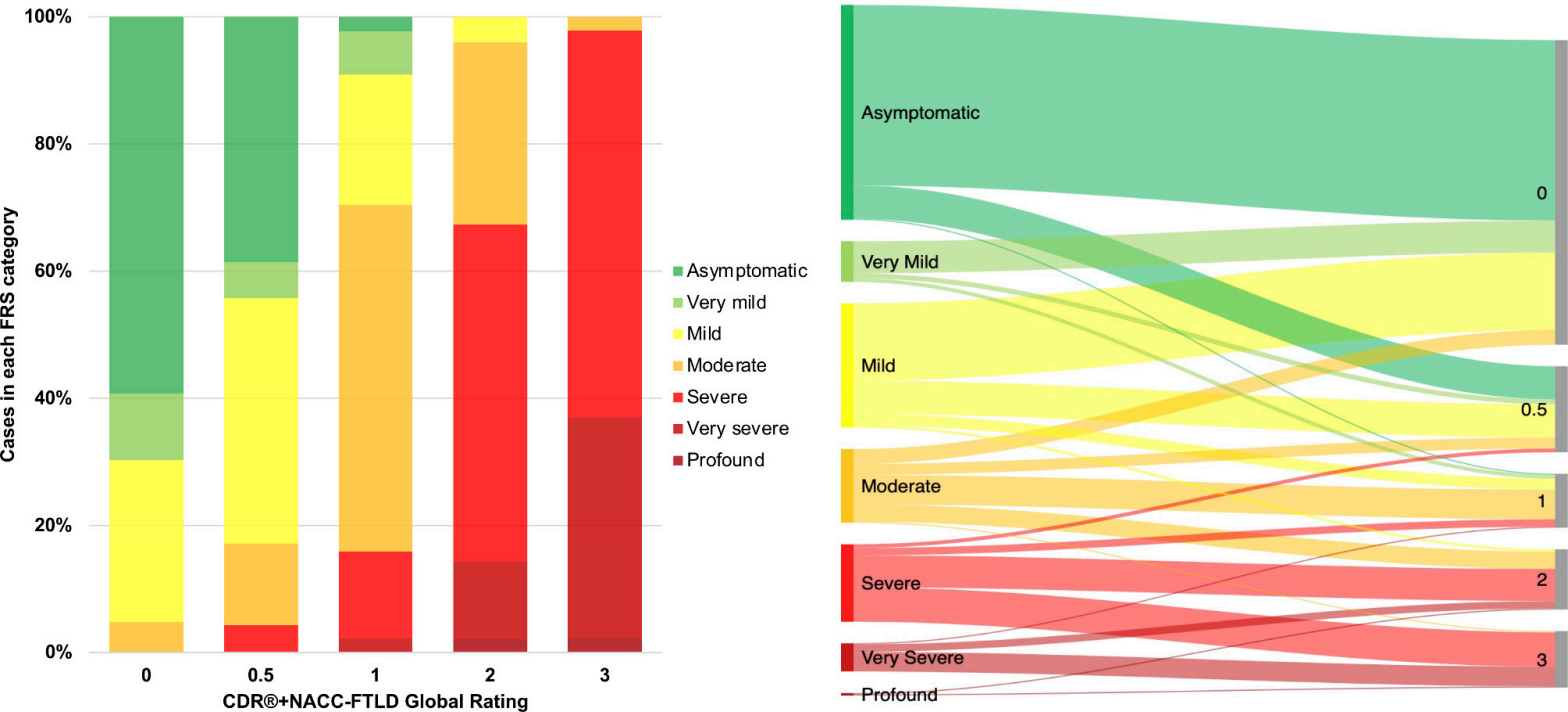
Bar graph and Sankey diagram presenting proportions of participants in each FRS severity category according to CDR+NACC FTLD global rating, in all mutation carriers at baseline. CDR+NACC FTLD global rating 0, N=248; 0.5, N=70; 1, N=44; 2, N=49; 3, N=46. CDR, Clinical Dementia Rating; FRS, Frontotemporal dementia Rating Scale; NACC, National Alzheimer’s Disease Coordinating Center.

The percentage of mutation carriers in each FRS severity category according to their CDR+NACC FTLD global rating, and vice versa, are shown in figure 3 (and individually for *GRN, MAPT* and *C9orf72* mutation carriers in online supplemental figure 2). Mutation carriers who had an FRS score in the ‘asymptomatic’ range most frequently had a global rating of 0 (84.0%); cases in the ‘very mild’ FRS category also predominantly had a global rating of 0 (78.8%); the ‘mild’ category encompassed cases with mostly global ratings of 0 (62.4%) or 0.5 (26.7%); the ‘moderate’ category covered cases with global ratings of 0 (20.0%), 0.5 (15.0%), 1 (40.0%) and 2 (23.3%); the ‘severe’ category mostly included ratings of 2 (41.3%) or 3 (44.4%); the ‘very severe’ category encompassed ratings of largely 3 (69.6%) as well as 2 (26.1%); and of the two participants who had an FRS score in the ‘profound’ range, one had a global rating of 2 (50%) and the other 3 (50%).

#### Frequency and severity of individual CDR+NACC-FTLD domains within each FRS severity category

**Figure 4:**
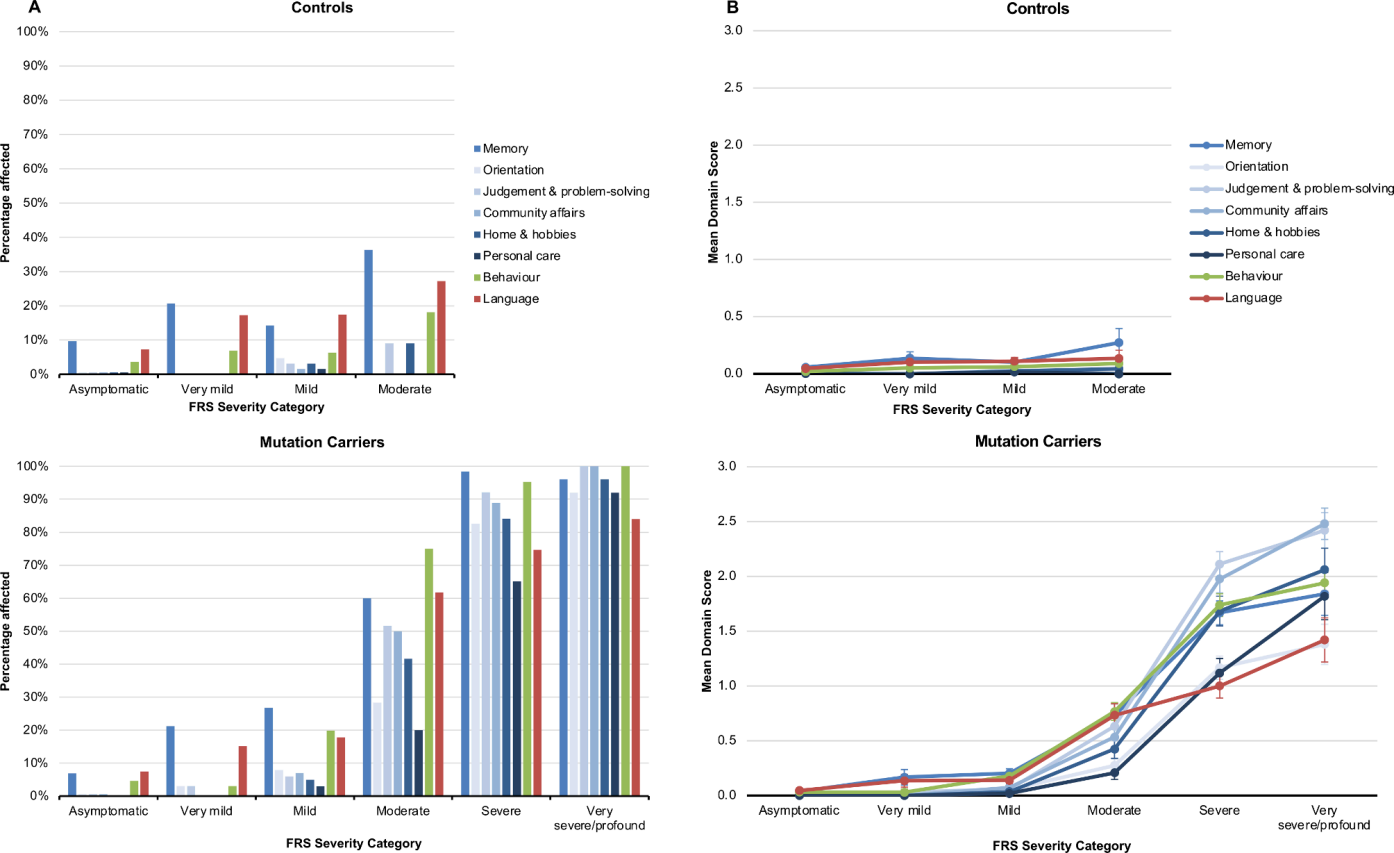
(A) Frequencies of CDR+NACC FTLD domains affected (rating ≥0.5) within each FRS severity category, in mutation carriers and non-carrier controls at baseline. FRS severity categories: controls: asymptomatic, N=165; very mild, N=29; mild, N=63; moderate, N=11; severe, N=0; very severe, N=0; profound, N=0; mutation carriers: asymptomatic, N=175; very mild, N=33; mild, N=101; moderate, N=60; severe, N=63; very severe, N=23; profound, N=2. Note that very severe and profound groups were combined for the mutation carriers due to limited cases in the profound group. (B) Mean scores on CDR+NACC FTLD domains within each FRS severity category, in mutation carriers and in non-carrier controls at baseline. Error bars represent SE of the mean. domains are rated using scores of 0 (absent), 0.5 (questionable/very mild), 1 (mild), 2 (moderate) and 3 (severe). CDR, Clinical Dementia Rating; FRS, Frontotemporal dementia Rating Scale; NACC, National Alzheimer’s Disease Coordinating Center.

The frequency of abnormal ratings (≥0.5) on the individual domains of the CDR+NACC FTLD within each FRS severity level are shown in figure 4A for mutation carriers and controls, and for the individual genetic groups in online supplemental figure 3A. Memory was the most commonly affected domain in the asymptomatic, very mild and mild FRS severity levels in both non-carriers and carriers.

The mean ratings for the CDR+NACC FTLD domains (ie, the severity) in each of the FRS levels are shown in figure 4B for mutation carriers and controls, and for the individual genetic groups in online supplemental figure 3B. Comparing the mean domain score of mutation carriers at each FRS stage against the mean score in controls for that domain: in the asymptomatic and very mild FRS stages, none of the domains were different from controls; in the mild stage, the memory (p=0.009), community affairs (p=0.040) and behaviour (p=0.002) domains had higher ratings than controls; and in the moderate, severe and very severe/profound FRS stages, all of the CDR+NACC FTLD domains had more severe ratings than controls.

#### Correlation of both FRS and CDR+NACC-FTLD-SB with other measures of disease severity

The FRS score was moderately negatively correlated with disease duration in symptomatic participants except in *C9orf72* mutation carriers (all symptomatic participants r_s_=−0.383, p<0.001; *GRN* r_s_=−0.541, p<0.001; *MAPT* r_s_=−0.525, p=0.008; *C9orf72* r_s_=−0.201, p=0.078), and positively correlated with MMSE score (all mutation carriers r_s_=0.614, p<0.001; *GRN* r_s_=0.654, p<0.001; *MAPT* r_s_=0.623, p<0.001; *C9orf72* r_s_=0.558, p<0.001).

The CDR+NACC-FTLD-SB score was also moderately positively correlated with increased disease duration in symptomatic participants (all symptomatic participants r_s_=0.426, p<0.001; *GRN* r_s_=0.518, p<0.001; *MAPT* r_s_=0.529, p=0.008; *C9orf72* r_s_=0.330, p=0.003), and negatively correlated with MMSE score (all mutation carriers r_s_=−0.646, p<0.001; *GRN* r_s_=−0.638, p<0.001; *MAPT* r_s_=−0.685, p<0.001; *C9orf72* r_s_=−0.618, p<0.001).

#### Comparison of both FRS and CDR+NACC-FTLD-SB with clinician judgment of diagnosis

The number of participants judged to be symptomatic by clinicians was 152: 103 bvFTD^7^ (27 *GRN*, 21 *MAPT*, 55 *C9orf72*), 24 PPA^8^ (20 *GRN*, 1 *MAPT*, 3 *C9orf72*), 16 ALS or FTD-ALS^24^ (all *C9orf72*), 4 with parkinsonian disorders^10 11^ (2 *GRN*, 1 *MAPT*, 1 *C9orf72*), and five diagnosed with a dementia not otherwise specified (1 *GRN*, 1 *MAPT*, 3 *C9orf72*).

The distributions of these diagnoses across the FRS severity categories and CDR+NACC FTLD global rating groups are shown in online supplemental table 5. Both rating scales classified four participants who had been judged as symptomatic within the lowest severity category (asymptomatic for FRS: 2 bvFTD, 1 PPA, 1 ALS/FTD-ALS; 0 for CDR+NACC-FTLD: 1 bvFTD, 2 ALS/FTD-ALS, 1 with a parkinsonian disorder). With increasing FRS severity and CDR+NACC FTLD global rating, an increasingly larger number of participants were judged to be symptomatic: by FRS severity – very mild 6.5%, mild 9.8%, moderate 60.6%, severe 95.2%, very severe/profound 100.0%; by CDR+NACC FTLD global rating – 0.5 16.2%, 1 70.0%, 2 98.0%, 3 100.0%.

### Longitudinal change in the FRS and CDR+NACC-FTLD

**Figure 5:**
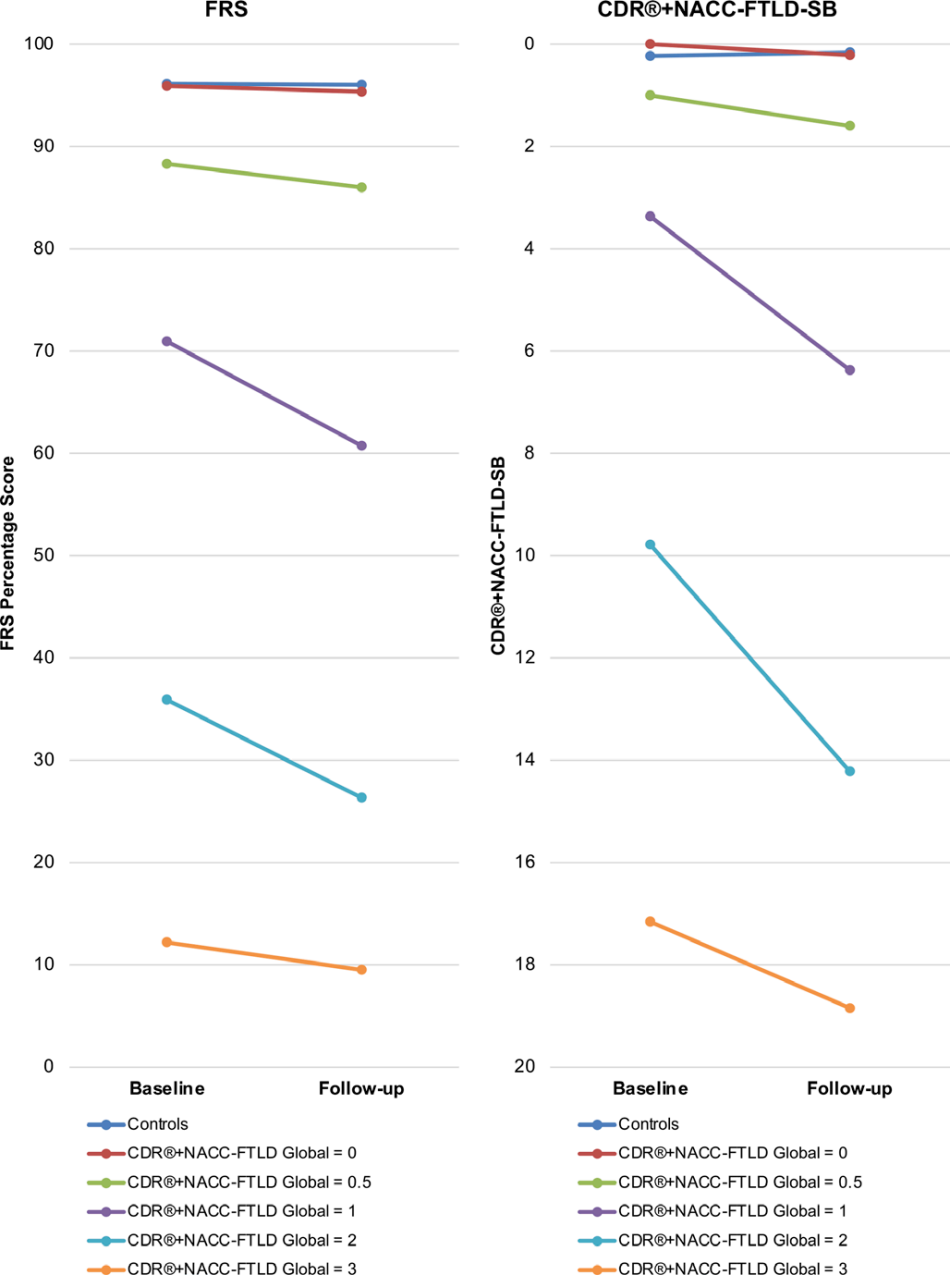
Annualised change in FRS percentage score and CDR+NACC FTLD sum of boxes score in mutation carriers according to baseline CDR+NACC FTLD global rating and controls. Baseline values=mean score, follow-up values=(baseline mean score)+(mean annualised change in score). Controls N=145; carriers N=232 (baseline CDR+NACC FTLD global 0, N=140; 0.5, N=30; 1, N=22; 2, N=23; 3, N=16). CDR, Clinical Dementia Rating; FRS, Frontotemporal dementia Rating Scale; NACC, National Alzheimer’s Disease Coordinating Center.

Annualised change on the FRS and CDR+NACC-FTLD-SB in controls and according to baseline CDR+NACC FTLD severity level in mutation carriers are reported in table 3 and online supplemental table 6 and shown in figure 5. Annualised change in FRS and CDR+NACC-FTLD-SB scores initially increased with global severity level and was greatest in carriers with symptomatic baseline global ratings of 1 or 2 before decreasing in cases with a global rating of 3. Mutation carriers with global ratings of 1 or 2 were the only groups to show change on the FRS over 1 year that significantly exceeded controls (p=0.011 and 0.005, respectively). On the CDR+NACC FTLD, annualised change in the Sum of Boxes score exceeded controls in each of the global severity levels except in the 0.5 group (0 group vs controls, p=0.001, 0.5 group vs controls, p=0.202, 1 group vs controls, p=0.001, 2 group vs controls (p<0.001, 3 group vs controls, p=0.033).

**Table 3:** Annualised change in FRS percentage and CDR+NACC FTLD sum of boxes scores in mutation carriers according to baseline CDR+NACC FTLD global rating, and non-carrier controls

CDR+NACC FTLD global rating at baseline	Mutation carriers					Controls
	0	0.5	1	2	3	
N	140	30	22	23	16	145
FRS % Score						
Annualised change	−0.5 (8.2)	−2.3 (9.9)	−10.2 (18.6)*, †	−9.6 (16.6)*, †	−2.7 (8.3)	−0.1 (6.0)
CDR+NACC-FTLD-SB						
Annualised change	0.2 (0.9)*	0.6 (2.7)	3.0 (4.1)*, †, ‡	4.4 (4.0)*, †, ‡, §	1.7 (3.3)*	−0.1 (0.4)

Values are mean (SD) unless stated.

CDR+NACC FTLD=CDR Dementia Staging Instrument plus Behaviour and Language domains from the NACC FTLD module.

*P<0.05 versus controls

†P<0.05 versus baseline CDR+NACC FTLD global rating=0

‡P<0.05 versus baseline CDR+NACC FTLD global rating=0.5

§P<0.05 versus baseline CDR+NACC FTLD global rating=3.

CDR, Clinical Dementia Rating; FRS, Frontotemporal dementia Rating Scale; FTLD, Frontotemporal Lobar Degeneration; NACC, National Alzheimer’s Disease Coordinating Center.

### Sample size calculations

Table 4 shows the number of participants required to demonstrate efficacy on change in FRS percentage score and CDR+NACC FTLD Sum of Boxes score as potential outcome measures when assuming small (10%) to moderate (30%) effect sizes. For a trial entering prodromal mutation carriers (with a global rating of 0.5), over 180 participants per arm would be needed to detect a moderate sized effect (30%) for both outcome measures. Power calculations using the FRS yielded projected sample sizes that were more favourable than the CDR+NACC FTLD Sum of Boxes.

**Table 4:** Sample sizes for small to moderate effect sizes on progression in mean FRS percentage score and mean CDR+NACC FTLD Sum of Boxes score, in mutation carriers with a global rating of 0.5

	Effect size on progression in mean score				
	30%	25%	20%	15%	10%
FRS percentage score					
N (per arm)	181	261	408	725	1630
CDR+NACC FTLD Sum of Boxes score					
N (per arm)	329	474	740	1315	2960

CDR+NACC FTLD=CDR Dementia Staging Instrument plus Behaviour and Language domains from the NACC FTLD module.

CDR, Clinical Dementia Rating; FRS, Frontotemporal dementia Rating Scale; FTLD, Frontotemporal Lobar Degeneration; NACC, National Alzheimer’s Disease Coordinating Center.

As the treatment effect is based on preventing progression from global CDR+NACC FTLD 0.5–1, the length of the trial depends on the natural history of this progression. A survival analysis in the GENFI cohort previously showed that ~50% of mutation carriers progress from a global rating of 0.5–1 in 3 years (Poos *et al* in submission). Against this background, a 6-year trial of prodromal mutation carriers would therefore be required to detect the proposed treatment effect (eg, for a 30% effect on FRS, N=181), *or* a 3-year trial of the same treatment would require the sample size equivalent to assuming half the percentage change in the target value (eg, if there was a 30% effect on FRS, as only 50% of people will have progressed, the sample size would be equivalent to a 15% effect on FRS that is, N=725).

## Discussion

This study has systematically evaluated and compared disease staging and progression defined by the FRS against the widely used CDR+NACC FTLD scale in a large cohort covering the spectrum of genetic FTD. Scores on both scales are strongly related to disease severity in FTD, and in *GRN, C9orf72* and *MAPT* mutation carriers, FRS scores decreased with progression while CDR+NACC-FTLD-SB increased. In direct comparison, both scores were strongly correlated with each other in all three genetic groups.

However, disease staging and severity were not entirely consistent between the two scales. Analysis indicated that the FRS might capture more subtle changes associated with disease progression. A notable proportion of cases were asymptomatic according to the CDR+NACC FTLD (zero cognitive, behavioural or language impairments recorded) despite a mild or moderate degree of functional and/or behavioural change being reported via the FRS questionnaire. Vice versa, a number of cases with a global rating of 0.5, or in a small number a rating of 1, scored 100% on the FRS (indicating zero behavioural or functional changes). In line with previous studies,^22 23^ our data suggest that the CDR+NACC FTLD may be more likely to underestimate disease severity when compared with FRS scores: 41% of cases with an asymptomatic CDR+NACC FTLD global rating had a degree of disability or behavioural change according to the FRS, vs 16% of cases with an asymptomatic FRS score having some symptomatology according to the CDR+NACC FTLD. Although the scales both broadly centre around everyday functioning and behaviour, there are differences between them e.g. the CDR+NACC FTLD evaluates language impairment, which the FRS lacks, but conversely, the CDR+NACC FTLD may not as comprehensively capture other changes apparent to the caregiver, for example, behaviour is captured as a single domain in the CDR+NACC FTLD which may underestimate social and personality impairments that rely on subjective report and are difficult to operationalise. Another consideration is that several of the activities of daily living probed by the FRS have the potential to be affected by apathy or depression (four items begin with ‘Lacks interest in… (activity)’), which are symptoms less relevant to the domains of the CDR+NACC FTLD. Whether depressive symptoms are directly related to evolving FTD pathology or are distinct and related to the impact of living at-risk of FTD is challenging to disentangle. Responses to the individual items of the FRS questionnaire were not available in the GENFI cohort to enable exploring trends among the cases with discrepant FRS and CDR+NACC FTLD scores, but this is a consideration for future studies of the scales.

There were also discrepancies seen between both scales and symptomatic status, with a small number of participants being judged to be symptomatic by clinicians despite an asymptomatic or very mild score on the two scales. This may relate at least in part to a further issue with both scales, which is the lack of an assessment for motor or neuropsychiatric symptoms. Parkinsonian symptoms are seen across all of the genetic forms of FTD,^25^ while ALS is seen mainly in those with *C9orf72* mutations. Such motor deficits are associated with disease progression,^26^ and impact on function in genetic FTD but are poorly captured by the FRS^27^ and not measured at all in the CDR+NACC FTLD. In this cohort, half of the participants diagnosed with ALS/FTD-ALS were in the asymptomatic, very mild or mild FRS severity categories or had asymptomatic (0) or very mild (0.5) CDR+NACC FTLD global ratings. Similarly, neuropsychiatric symptoms are also prevalent across the different forms of genetic FTD,^28 29^ particularly in carriers of the *C9orf72* expansion where they can be a defining feature.^30 31^ Neither of the scales directly measure these features (ie, hallucinations, delusions, etc) and therefore are likely to be underestimating any effect of such symptoms on function and disease progression. Overall, given the heterogeneity in clinical presentation and disease course within people that share the same underlying genetic cause,^12^ the inclusion of assessments of motor and neuropsychiatric symptomatology into clinical rating scales will be important for achieving accurate evaluation of disease stage. In turn, this will allow the full spectrum of FTD phenotypes to be included within the same clinical trial.

To evaluate the scale’s abilities to track progression, annualised change was analysed in the cases with a follow-up time point, stratified by global impairment at baseline according to the CDR+NACC FTLD. On both scales, change over 1 year is small in the prodromal stages and then accelerates in carriers with a global rating considered to be symptomatic. Previous studies have reported significant changes in CDR+NACC FTLD scores over 1^15^ and 2 years^20^ in patients with FTD. We found that annualised change also accelerated moving from an asymptomatic global rating to a very mild 0.5 rating, and moving from 0.5 to 1. Our data align with previous findings that the FRS is able to detect deterioration over 1 year in symptomatic patients,^21^ and show that this is the case particularly in those with ‘mild’ and ‘moderate’ FTD defined by the CDR+NACC FTLD global score.

Lastly, we estimated the sample sizes required to achieve at least 80% power to detect small to moderate sized effects of a disease-modifying therapy on change in the two scales as outcome measures. The sample sizes generated for both scales, even with a moderate (30%) treatment effect, suggest that a trial entering mutation carriers at a prodromal starting point (of CDR+NACC FTLD global rating 0.5) in an unselective way (ie, that does not further distinguish cases that are likely to soon progress) will require large numbers (with even greater numbers being required if randomisation was unequal rather than 1:1) and several years. The period in close proximity to phenoconversion is a useful target period for disease-modifying therapies, but for such a trial to require achievable sample sizes, this study suggests that better stratification will be needed, potentially combining clinical stage with neuroanatomical and/or fluid biomarkers to accurately identify likely converters. For example, a study involving GENFI and another genetic FTD cohort has recently shown that mutation carriers whose score worsens on the CDR+NACC FTLD over the next 1–2 years have high plasma neurofilament light chain concentrations at baseline compared with non-converters.^32^

### Limitations

By including a large number of mutation carriers at varied proximities to symptom onset, this study was able to evaluate the utility of disease staging tools across the spectrum of genetic FTD. However, the study cohort at baseline contains a larger proportion of asymptomatic than symptomatic carriers, and once stratified, individual group numbers were smaller. We took a transdiagnostic approach to the study, incorporating all phenotypes in the analysis. We, therefore, did not establish whether the scales were better at evaluating one phenotype over the other, although this is difficult as our study contained mainly people with a bvFTD phenotype (as is the case for genetic FTD), and few with PPA or FTD-ALS.^27^ We were also not able to directly assess the ability of the scales to specifically measure the presence of prodromal symptoms as we did not have another marker of this stage, for example, clinician judgment. As discussed above, it may be that both scales (but particularly the CDR+NACC FTLD) are not sensitive enough to adequately capture this stage, and further studies should try to address this point.

## Conclusions

Global rating scales such as the CDR+NACC FTLD and FRS serve a helpful purpose in clinical trials in providing a single score that can condense clinical judgement about disease severity. Although the CDR+NACC FTLD has become the most prominent clinical rating scale in FTD, there are potential issues with its use in clinical trials. In this study we show that there are similarities to the FRS as well as differences, and highlight the potential benefits for using the FRS both in clinical stratification and as an outcome measure in prevention trials of genetic FTD mutation carriers. However, both measures do not fully capture the entire spectrum of FTD symptomatology, and future improvements to the scales should consider the inclusion of motor and neuropsychiatric deficits.

## Data Availability

Data are available on reasonable request. Anonymised participant data are held by GENFI and available upon reasonable request from JDR, j.rohrer@ucl.ac.uk.
