# Supplementary material for: Comparison of clinical rating scales in genetic frontotemporal dementia within the GENFI cohort
Source: J Neurol Neurosurg Psychiatry. 2021 Aug 5;93(2):158–68. doi: 10.1136/jnnp-2021-326868 (PMC8785074; doi:10.1136/jnnp-2021-326868)

Supplementary Table 1. Adjusted mean differences with 95% confidence intervals and p-values for the linear regression model comparing FRS percentage score between groups. Significant differences are shown in bold.

	***GRN* mutation carriers**		***MAPT* mutation carriers**		***C9orf72* mutation carriers**	
**Controls**	**-12.8**		**-18.0**		**-25.2**	
	**-17.4**	**-8.2**	**-24.3**	**-11.8**	**-29.7**	**-20.6**
	**<0.001**		**<0.001**		**<0.001**	
***GRN* mutation carriers**			-5.2		**-12.3**	
			-11.8	1.3	**-17.3**	**-7.4**
			0.117		**<0.001**	
***MAPT* mutation carriers**					**-7.1**	
					**-13.6**	**-0.6**
					**0.032**	

Supplementary Table 2. Adjusted mean differences with 95% confidence intervals and p-values for the linear regression model comparing CDR®+NACC-FTLD Sum of Boxes between groups. Significant differences are shown in bold.

	***GRN* mutation carriers**		***MAPT* mutation carriers**		***C9orf72* mutation carriers**	
**Controls**	**2.4**		**2.9**		**3.9**	
	**1.5**	**3.2**	**1.8**	**4.1**	**3.1**	**4.8**
	**<0.001**		**<0.001**		**<0.001**	
***GRN* mutation carriers**			0.5		**1.5**	
			-0.7	1.8	**0.6**	**2.4**
			0.373		**0.001**	
***MAPT* mutation carriers**					1.0	
					-0.2	2.2
					0.113	

Supplementary Table 3. Adjusted mean differences with 95% confidence intervals and p-values for the linear regression model comparing FRS percentage score by disease severity within genetic groups. Significant differences are shown in bold.

a. *GRN* mutation carriers

	**0**		**0.5**		**≥1**	
**Controls**	1.2		-5.3		**-49.9**	
	-0.2	2.5	-11.1	0.6	**-58.1**	**-41.8**
	0.104		0.078		**<0.001**	
**0**			**-6.4**		**-51.1**	
			**-12.3**	**-0.5**	**-59.2**	**-43.0**
			**0.033**		**<0.001**	
**0.5**					**-44.6**	
					**-54.4**	**-34.9**
					**<0.001**	

b. *MAPT* mutation carriers

	**0**		**0.5**		**≥1**	
**Controls**	-3.2		-6.5		**-53.2**	
	-6.7	0.3	-16.9	3.9	**-64.6**	**-41.9**
	0.075		0.219		**<0.001**	
**0**			-3.3		**-50.1**	
			-14.2	7.5	**-62.0**	**-38.1**
			0.547		**<0.001**	
**0.5**					**-46.7**	
					**-62.3**	**-31.2**
					**<0.001**	

c. *C9orf72* mutation carriers

	**0**		**0.5**		**≥1**	
**Controls**	-1.3		**-11.2**		**-63.5**	
	-3.2	0.6	**-18.4**	**-4.0**	**-69.3**	**57.6**
	0.186		**0.002**		**<0.001**	
**0**			**-9.9**		**-62.2**	
			**-17.2**	**-2.6**	**-68.3**	**56.1**
			**0.008**		**<0.001**	
**0.5**					**-52.3**	
					**-61.4**	**-43.1**
					**<0.001**	

Supplementary Table 4. Adjusted mean differences with 95% confidence intervals and p-values for the linear regression model comparing CDR®+NACC-FTLD Sum of Boxes by disease severity within genetic groups. Significant differences are shown in bold.

a. *GRN* mutation carriers

	0		0.5		≥1	
Controls	-0.2		0.8		9.5	
	-0.3	-0.2	0.5	1.1	7.7	11.2
	<0.001		<0.001		<0.001	
0			1.0		9.7	
			0.7	1.3	8.0	11.5
			<0.001		<0.001	
0.5					8.7	
					6.9	10.5
					<0.001	

b. *MAPT* mutation carriers

	0		0.5		≥1	
Controls	-0.2		0.9		10.2	
	-0.3	-0.2	0.4	1.3	7.8	12.7
	<0.001		<0.001		<0.001	
0			1.1		10.5	
			0.7	1.5	8.0	12.9
			<0.001		<0.001	
0.5					9.4	
					6.9	11.8
					<0.001	

c. *C9orf72* mutation carriers

	0		0.5		≥1	
Controls	-0.2		0.9		10.9	
	-0.3	-0.2	0.6	1.2	9.6	12.1
	<0.001		<0.001		<0.001	
0			1.1		11.1	
			0.9	1.4	9.8	12.4
			<0.001		<0.001	
0.5					10.0	
					8.6	11.3
					<0.001	

Supplementary Table 5. Percentages of participants judged as symptomatic by clinicians within both the FRS severity categories and CDR®+NACC-FTLD global rating groups at baseline. CDR®+NACC-FTLD = Clinical Dementia Rating (CDR®) Dementia Staging Instrument plus Behavior and Language domains from the National Alzheimer's Disease Coordinating Center Frontotemporal Lobar Degeneration module. FRS = Frontotemporal dementia Rating Scale. bvFTD = behavioural variant frontotemporal dementia, PPA = primary progressive aphasia, ALS = amyotrophic lateral sclerosis, FTD-ALS = frontotemporal dementia with amyotrophic lateral sclerosis, dementia-NOS = dementia not otherwise specified.

	Total N	All diagnoses	bvFTD	PPA	ALS/FTD-ALS	Parkinsonian disorders	Dementia-NOS
**FRS severity categories**							
Asymptomatic	**340**	4 (1.2)	2 (0.6)	1 (0.3)	1 (0.3)	0 (0.0)	0 (0.0)
Very mild	**62**	4 (6.5)	0 (0.0)	1 (1.6)	1 (1.6)	2 (3.2)	0 (0.0)
Mild	**164**	16 (9.8)	7 (4.3)	2 (1.2)	5 (3.0)	0 (0.0)	2 (1.2)
Moderate	**71**	43 (60.6)	26 (36.6)	13 (18.3)	3 (4.2)	0 (0.0)	1 (1.4)
Severe	**63**	60 (95.2)	47 (74.6)	5 (7.9)	5 (7.9)	2 (3.2)	1 (1.6)
Very severe /profound	**25**	25 (100.0)	21 (84.0)	2 (8.0)	1 (4.0)	0 (0.0)	1 (4.0)
**CDR®+NACC-FTLD global rating**							
0	**463**	4 (0.9)	1 (0.2)	0 (0.0)	2 (0.4)	1 (0.2)	0 (0.0)
0.5	**117**	19 (16.2)	10 (8.6)	3 (2.6)	6 (5.1)	0 (0.0)	0 (0.0)
1	**50**	35 (70.0)	21 (42.0)	10 (20.0)	3 (6.0)	1 (2.0)	0 (0.0)
2	**49**	48 (98.0)	35 (71.4)	6 (12.2)	2 (4.1)	1 (2.0)	4 (8.2)
3	**46**	46 (100.0)	36 (78.3)	5 (10.9)	3 (6.5)	1 (2.2)	1 (2.2)

Supplementary Table 6. Adjusted mean differences with 95% confidence intervals and p-values for the linear regression model comparing annualised change in FRS percentage score and CDR®+NACC-FTLD Sum of Boxes. Significant differences are shown in bold.

a. *FRS*

	0		0.5		1		2		3	
Controls	-0.4		-2.2		**-10.1**		**-9.5**		-2.6	
	-2.2	1.3	-5.9	1.5	**-17.9**	**-2.3**	**-16.2**	**-2.8**	-6.8	1.6
	0.616		0.244		**0.011**		**0.005**		0.225	
0			-1.8		**-9.7**		**-9.0**		-2.1	
			-5.6	2.1	**-17.4**	**-1.9**	**-15.8**	**-2.3**	-6.4	2.1
			0.366		**0.015**		**0.009**		0.322	
0.5					-7.9		-7.3		-0.4	
					-16.4	0.6	-14.8	0.3	-5.8	5.0
					0.068		0.059		0.892	
1							0.6		7.5	
							-9.5	10.8	-1.2	16.2
							0.905		0.091	
2									6.9	
									-0.9	14.7
									0.081	

b. CDR®+NACC-FTLD-SB

	0		0.5		1		2		3	
Controls	**0.3**		0.6		**3.1**		**4.5**		**1.8**	
	**0.1**	**0.4**	-0.4	1.7	**1.3**	**4.8**	**2.9**	**6.1**	**0.1**	**3.4**
	**0.001**		0.202		**0.001**		**<0.001**		**0.033**	
0			0.4		**2.8**		**4.2**		1.5	
			-0.6	1.4	**1.1**	**4.5**	**2.6**	**5.9**	-0.1	3.1
			0.468		**0.002**		**<0.001**		0.073	
0.5					**2.4**		**3.9**		1.1	
					**0.4**	**4.4**	**2.0**	**5.7**	-0.8	3.0
					**0.018**		**<0.001**		0.259	
1							1.4		-1.3	
							-0.9	3.8	-3.7	1.1
							0.235		0.277	
2									**-2.7**	
									**-5.0**	**-0.5**
									**0.016**	

Supplementary Figure 1. Scatter plots of FRS percentage scores and CDR®+NACC-FTLD Sum of Boxes scores in GRN, MAPT and C9orf72 mutation carriers at baseline.

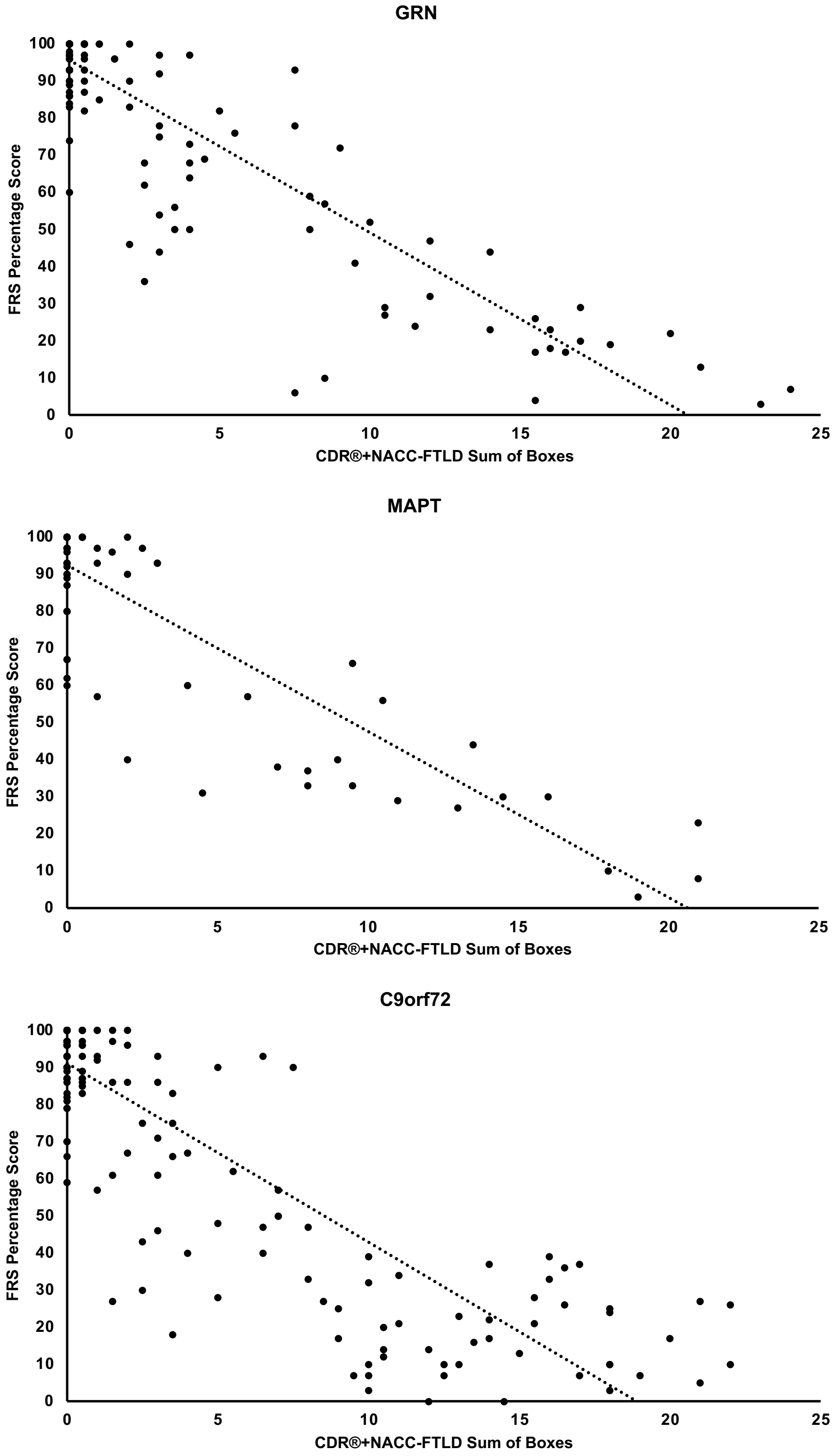

Supplementary Figure 2. Bar graphs and Sankey diagrams presenting proportions of participants in each FRS severity category according to CDR®+NACC-FTLD Global rating, in mutation carriers at baseline. CDR®+NACC-FTLD global ratings: GRN 0, N=114; 0.5, N=25; 1, N=19; 2, N=15; 3, N=14; MAPT 0, N=42; 0.5, N=13; 1, N=7; 2, N=8; 3, N=7; C9orf72 0, N=92; 0.5, N=32; 1, N=18; 2, N=26; 3, N=25.

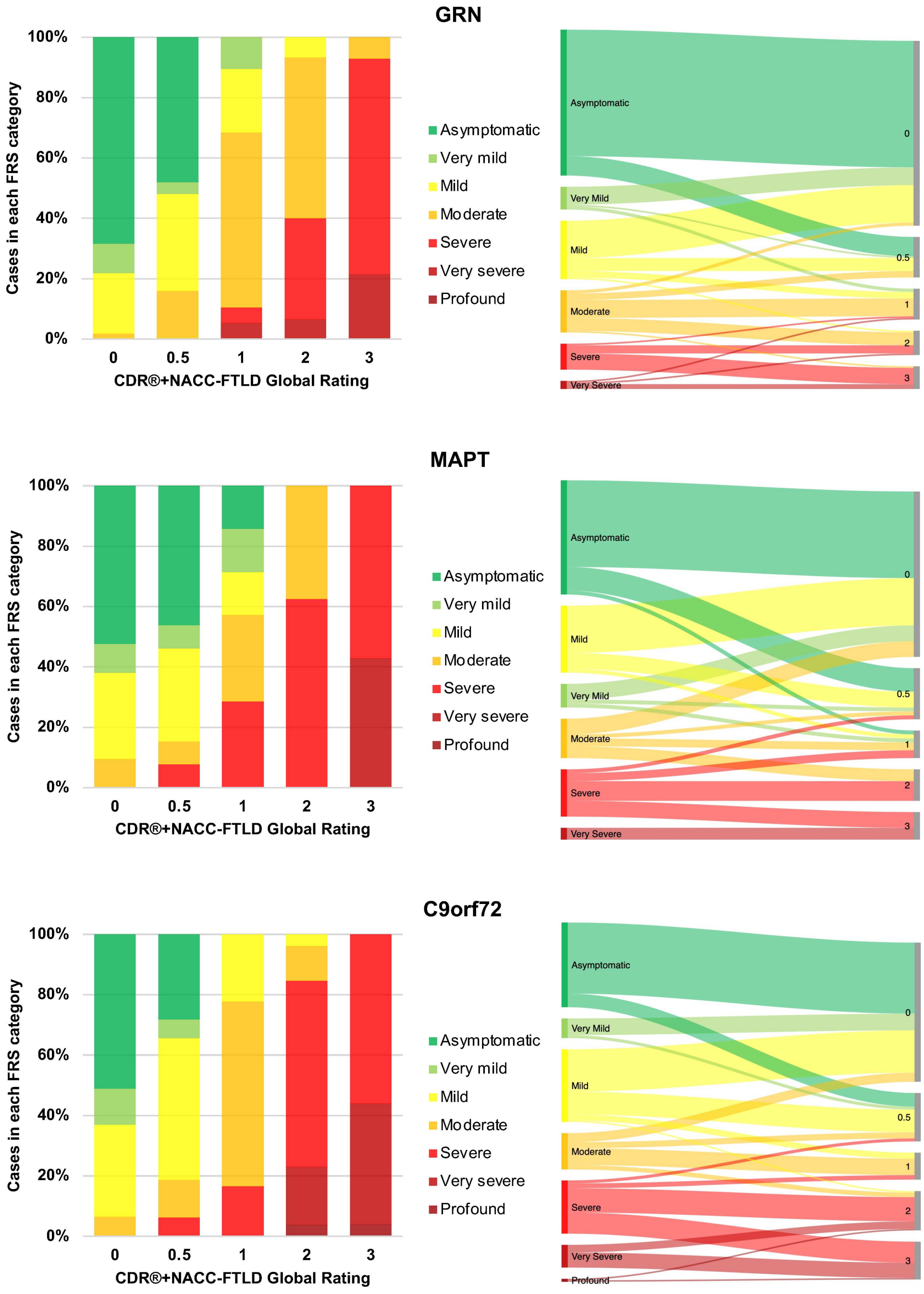

Supplementary Figure 3a. Frequencies of CDR®+NACC-FTLD domains affected (rating ≥0.5) within each FRS severity category in GRN, MAPT and C9orf72 mutation carriers at baseline. FRS severity categories: GRN: asymptomatic, N=90; very mild, N=14; mild, N=36; moderate, N=26; severe, N=16; very severe, N=5; profound, N=0. MAPT: asymptomatic, N=29; very mild, N=6; mild, N=17; moderate, N=10; severe, N=12; very severe, N=3; profound, N=0. C9orf72: asymptomatic, N=56; very mild, N=13; mild, N=48; moderate, N=24; severe, N=35; very severe, N=15; profound, N=2.

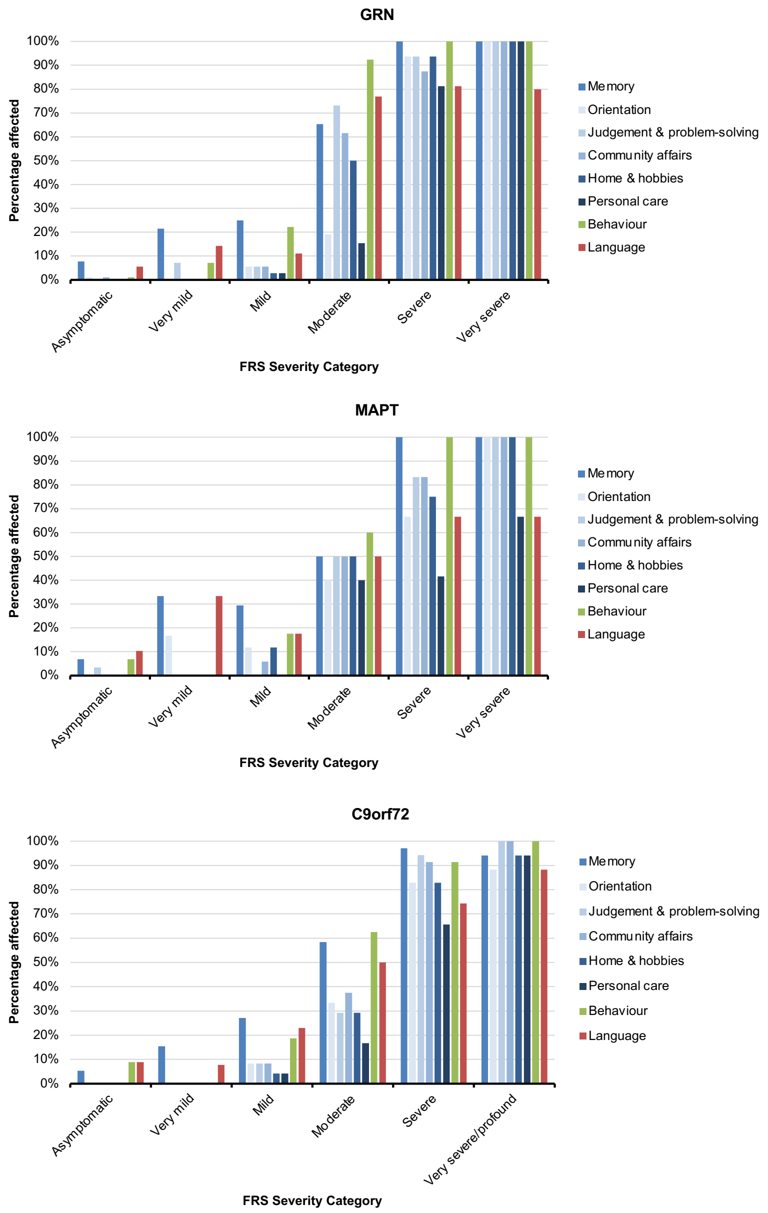

Supplementary Figure 3b. Mean scores on CDR®+NACC-FTLD domains within each FRS severity category in GRN, MAPT and C9orf72 mutation carriers at baseline. Error bars represent standard error of the mean. FRS severity categories: GRN: asymptomatic, N=90; very mild, N=14; mild, N=36; moderate, N=26; severe, N=16; very severe, N=5; profound, N=0. MAPT: asymptomatic, N=29; very mild, N=6; mild, N=17; moderate, N=10; severe, N=12; very severe, N=3; profound, N=0. C9orf72: asymptomatic, N=56; very mild, N=13; mild, N=48; moderate, N=24; severe, N=35; very severe, N=15; profound, N=2.

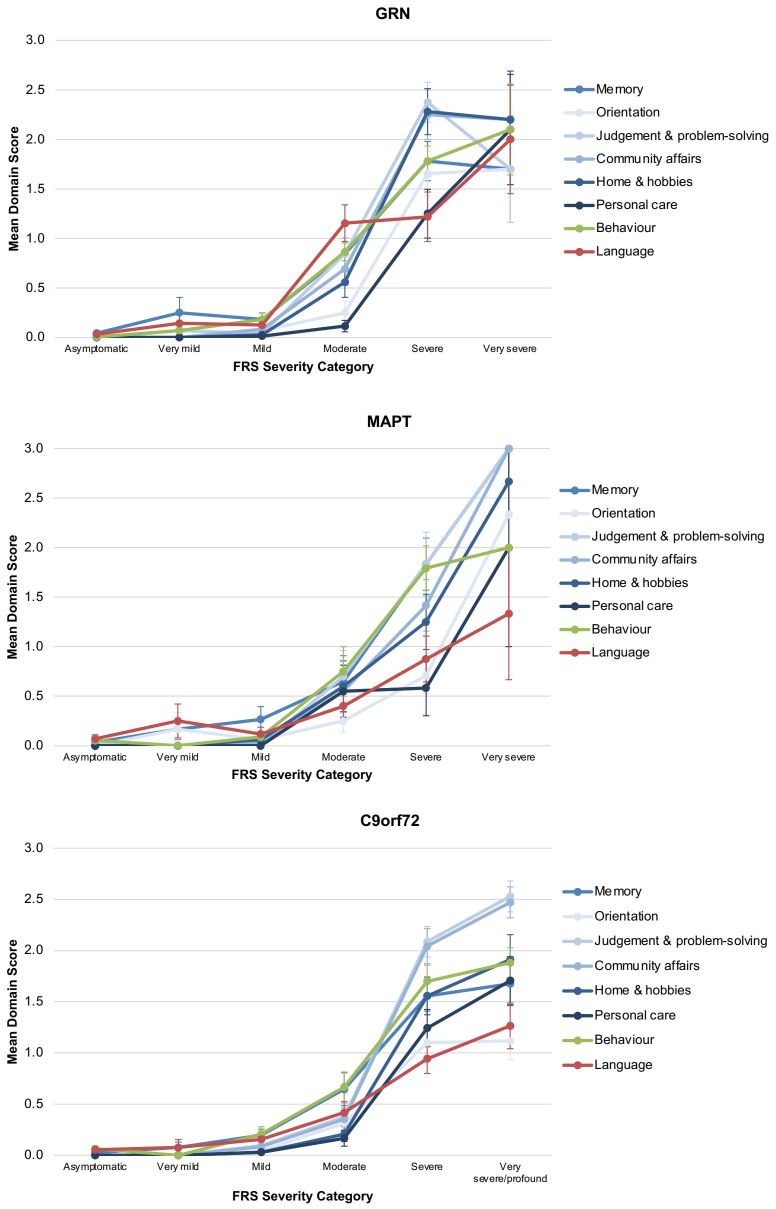

Supplement: Supplementary data [file jnnp-2021-326868supp001.pdf]
